# Functional Characterization of the *MsFKF1* Gene Reveals Its Dual Role in Regulating the Flowering Time and Plant Height in *Medicago sativa* L.

**DOI:** 10.3390/plants13050655

**Published:** 2024-02-27

**Authors:** Xu Jiang, Lili Zhang, Yajing Li, Ruicai Long, Qingchuan Yang, Junmei Kang

**Affiliations:** 1 Institute of Animal Science, Chinese Academy of Agricultural Sciences, Beijing 100193, China; jiangxu2009@yeah.net (X.J.); ly_yajing@yeah.net (Y.L.); dragongodsgod@163.com (R.L.);; 2 College of Life Science and Technology, Harbin Normal University, Harbin 150025, China

**Keywords:** alfalfa, FKF1, flowering time, circadian clock, blue light

## Abstract

Alfalfa (*M. sativa*), a perennial legume forage, is known for its high yield and good quality. As a long-day plant, it is sensitive to changes in the day length, which affects the flowering time and plant growth, and limits alfalfa yield. Photoperiod-mediated delayed flowering in alfalfa helps to extend the vegetative growth period and increase the yield. We isolated a blue-light phytohormone gene from the alfalfa genome that is an ortholog of soybean *FKF1* and named it *MsFKF1*. Gene expression analyses showed that *MsFKF1* responds to blue light and the circadian clock in alfalfa. We found that *MsFKF1* regulates the flowering time through the plant circadian clock pathway by inhibiting the transcription of *E1* and *COL*, thus suppressing *FLOWERING LOCUS T a1* (*FTa1*) transcription. In addition, transgenic lines exhibited higher plant height and accumulated more biomass in comparison to wild-type plants. However, the increased fiber (NDF and ADF) and lignin content also led to a reduction in the digestibility of the forage. The key genes related to GA biosynthesis, *GA20OX1*, increased in the transgenic lines, while *GA2OX1* decreased for the inactive GA transformation. These findings offer novel insights on the function of *MsFKF1* in the regulation of the flowering time and plant height in cultivated *M. sativa*. These insights into *MsFKF1*’s roles in alfalfa offer potential strategies for molecular breeding aimed at optimizing flowering time and biomass yield.

## 1. Introduction

Alfalfa (*Medicago sativa* L.), widely grown globally, is favored for its exceptional yield, superior quality, and perennial growth characteristics [1,2,3,4,5]. Improving agricultural production heavily depends on the efficient optimization of the flowering time in forage crops, which remains a prominent objective within breeding programs. Early flowering can help harvest seeds and avoid the rainy season [6]. However, early flowering is often correlated with lower biomass accumulation and forage quality. This is because the transition to flowering is accompanied by increased tissue lignification, stem-to-leaf tissue ratio, and the recycling of nutrients from the leaves to support seed development [4,7,8,9]. In high-latitude regions of China, where alfalfa is predominantly grown, its long-day growth habit (12–14 h of light duration) leads to early flowering [4]. Therefore, understanding the regulatory mechanisms that govern the flowering time in response to photoperiods is crucial for domestication breeding.

Photoperiodic flowering is regulated by the day length associated with the circadian clock in plant species [10]. Photoreceptors, such as phytochromes (PHY) and blue-light photoreceptor cryptochromes (CRY), play important roles in the transduction of light signals [11,12,13]. In addition to *PHY* and *CRY*, the circadian clock-controlled blue-light receptor FLAVIN-BINDING, KELCH REPEAT, and F-BOX 1 (FKF1) were shown to control flowering time [14]. Within the SKP1/CUL1/F-box (SCF)-type E3 ligase complex, FKF1 assumes a crucial role and encompasses three conserved domains: light, oxygen, voltage (LOV/PAS), F-box, and KELCH repeat. The unique structure of FKF1 enables it to mediate target protein degradation through the light-dependent ubiquitin proteasome pathway, thus regulating plant growth and development [15,16]. Blue light induces FKF1 to bind to the flavin mononucleotide chromophore through the LOV domain [17,18]. The F-box domain specifically recruits target proteins for ubiquitination and degradation, mainly through the formation of protein complexes [19]. The KELCH repeat domain, featuring multiple tandem repeats of the KELCH motif, adopts a β-propeller structure and plays a critical role in protein recruitment and degradation [19]. *FKF1* encoding genes have been found in a diverse array of higher plant species, encompassing not only model plants like *Arabidopsis* [20] but also a spectrum of crops, such as rice, soybeans, and tomato [21,22,23,24], as well as tree species [25,26]. Most of these *FKF1* genes have been studied using bioinformatics methods to understand their physical and chemical attributes, as well as their structural characteristics, and molecular and genetic techniques have been utilized to explore the biological roles of specific *FKF1* genes [21,22,24]. However, the function of *FKF1* in controlling flowering and growth in *Medicago* is unclear.

In the clearly defined *Arabidopsis* photoperiod pathway, the *CONTANS* (*CO*)—*flowering locus T* (*FT*) clade, regulated by the FKF1 protein, mediates flowering regulation in response to light signals. The florigen protein encoded by the *FT* gene has the ability to be transported via the phloem from the leaves to the shoot apical meristem and triggers the transition to flowering through activating the *SUPPRESSOR OF OVEREXPRESSION OF CONSTANS 1* (*SOC1*) [27]. Under long-day conditions, the *CO* gene encodes a zinc-finger-type transcription factor which directly binds to the *FT* promoter, thus activating transcription and promoting flowering [28]. FKF1 regulates the stability of the CO protein at the transcriptional and post-transcriptional levels. The *Cycling DOF factors* (CDF) transcription factor directly represses the expression of *CO* and *FT* by binding to its promoter [29,30]. In the afternoon of a long day, *FKF1* expression and light activate FKF1 protein activity, which forms a protein complex with GIGANTEA (GI) to degrade CDFs, thereby promoting *CO* and *FT* expression, leading to early flowering [30,31,32]. In contrast, the expression pattern of *FKF1* is shifted to expression after dusk in short-day conditions, and the loss of FKF1 affinity for GI results in the persistence of CDF, thereby inhibiting the transcription level of *CO* [32]. For post-transcriptional regulation, FKF1 maintains the stability of the CO protein by competitively binding with CONSTITUTIVELY PHOTOMORPHOGENIC 1 (COP1) and disrupting its self-dimerization, thereby inhibiting COP1-mediated degradation of the CO protein [33,34].

Research indicates that *FT* genes also play a conserved role in promoting flowering in legume plants, including temperate legumes, garden peas, and *Medicago* [1,4,35]. Despite the presence of five *FT* genes in the alfalfa genome, none of them share the circadian expression pattern observed in *Arabidopsis thaliana*, indicating a more intricate and sophisticated regulation mechanism for flowering in alfalfa [4,36]. However, the *M. truncatula* genome lacks *CO* orthologs, and *M. truncatula CO-like* (*COL*) genes seem to play a less crucial role in flowering regulation than *Arabidopsis thaliana* [37]. In alfalfa, it is the legume-specific gene *E1*, rather than *CO*, that plays a crucial role in regulating flowering time through the photoperiodic pathway. Under long-day conditions, mutant varieties of *M. truncatula* with *E1* gene alterations exhibit delayed flowering [38]. The *EC*–*E1*–*FT* pathway was reported to function in the control of photoperiod sensitivity in soybeans [39]. Recently, two homologs of soybean *GmFKF1s* were found to be genetically dependent on *E1*. They activate *E1* transcription by binding to its promoter, which subsequently represses the transcription of *FLOWERING LOCUS T 2a* (*FT2a*) and *FT5a* [22]. However, there are still gaps in our understanding of *FKF1*-mediated photoperiod flowering in alfalfa.

This study aimed to fill this gap by exploring the role of *FKF1* in *M. sativa*. We hypothesized that the *MsFKF1* gene, an ortholog of soybean *FKF1*, plays a significant role in regulating the flowering time and plant height in alfalfa. By functionally characterizing *MsFKF1* and examining its influence on key genetic pathways, this study sought to provide novel insights into the photoperiodic flowering mechanism in alfalfa and to identify potential targets for genetic manipulation to optimize the yield and quality in this crucial forage crop.

## 2. Results

### 2.1. MsFKF1 Encodes a Putative Photoreceptor Protein That Shares High Sequence Similarity with FKF1 Orthologs

Plant *FKF1* is a member of the ZTL/FKF1/LKP2 family, which includes two other members: ZEITLUPE (ZTL) and LOV KELCH PROTEIN 2 (LKP2). To identify *FKF1* orthologs in cultivar alfalfa, we performed a BLAST search using the *Arabidopsis* AtFKF1 (At1g68050) protein sequence as the query against the latest *M. sativa* genome database and found two similar genes (*Msa0930760* and *Msa1431740*), sharing 79.77% and 64.90% homology, respectively. Although they contain similar conserved domains, phylogenetic analysis of the two proteins showed that *Msa0930760* is more closely related to *Arabidopsis FKF1* and named *MsFKF1*, while *Msa1431740* is closely related to *Arabidopsis ZTL* (Figure S1).

**Figure 1:**
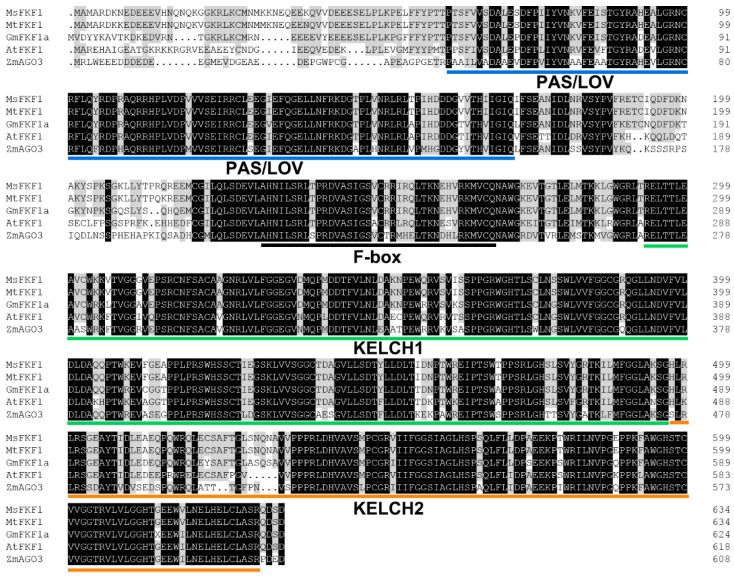
Alignment of MsFKF1 with its homologs from other species. Amino acid sequence alignment of FLAVIN-BINDING, KELCH REPEAT, and F-BOX 1 (FKF1) in *Medicago sativa* (MsFKF1), *M. truncatula* (MtFKF1), *Glycine max* (GmFKF1), *Arabidopsis thaliana* (AtFKF1), and *Zea mays* (ZmAGO3). Identical amino acids are indicated by a black background, while the gray shade represents amino acids that share a similarity of 50% or more. PAS/LOV (blue), F-box (black), and the KELCH repeat domain (green and orange) are marked in continuous underline, according to the InterPro website tool.

The *MsFKF1* gene was cloned and sequence analysis of the 1979 bp fragment revealed a predicted ORF of 1939 bp that encodes a polypeptide comprising 645 amino acids (Figure 1). The predicted MsFKF1 protein has an estimated molecular weight of approximately 71.03 kDa and a theoretical isoelectric point of 6.41. Multiple sequence alignment illustrated a high sequence similarity between *MsFKF1* and its homologous genes (72.9–99.3%) (Table S1). Further analysis suggested that this is attributed to three conserved functional domains: LOV, F-BOX, and KELCH repeat sequences (Figure 1). Analysis of the *FKF1* gene structure in different species indicated that the *FKF1* gene in higher plants is highly conserved, comprising two exons and one intron (Table S2). Based on the phylogenetic analysis (Figure S2), it was evident that the *FKF1* homologous genes in various plant species exhibited distinct evolutionary patterns, with a clear separation into two major branches representing monocotyledonous and dicotyledonous plants. The MsFKF1 protein is located in a branch of the latter and clusters with *FKF1* homologous genes in the Fabaceae subfamily, including alfalfa and soybean.

### 2.2. Expression Analysis of MsFKF1 in Alfalfa Tissues and Mainly Located in the Nucleus

**Figure 2:**
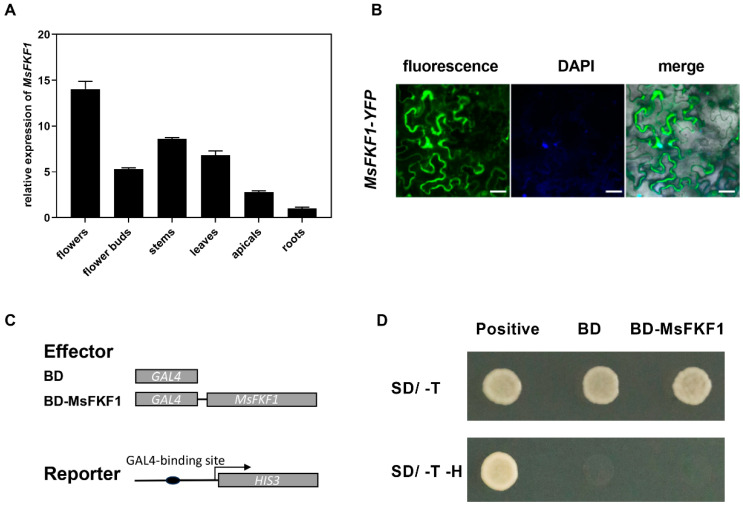
Analysis of the expression characteristics of *MsFKF1*. (**A**) The expression levels of *MsFKF1* in different tissues. RNA was extracted from the roots, stems, leaves, buds, and flowers of WT plants grown under LD conditions for 45 days. The results represent the average of three independent biological replicates, and the error bars indicate the standard deviation. (**B**) Subcellular localization of the MsFKF1-GFP fusion protein in the tobacco epidermal layers analyzed by fluorescence microscopy. The fluorescence of GFP, DAPI, and merged images are shown. DAPI represent 4′,6-diamidino-2-phenylindole (nucleus-specific dye). Scale bar = 50 μm. (**C**) Schematic diagram of the analysis of the transcriptional activation activity of *MsFKF1*. (**D**) The *MsFKF1* gene lacks transcriptional activation activity in yeast (Y2H Gold). SD/-T refers to synthetic dropout (SD) yeast growth medium that is deficient in tryptophan (-T). SD/-L-H, SD medium without tryptophan and histidine, and supply with 15 mM 3-AT. Positive means the co-transition of yeast with PGBKT7-53 and PGADT7-T, which could activate the *HIS3* reporter gene. BD and BD-MsFKF1 indicate the PGBKT7 vector and PGBKT7 fused with *MsFKF1*, respectively.

RT-qPCR was performed to determine the expression pattern of *MsFKF1* in alfalfa tissues. *MsFKF1* showed a ubiquitous expression pattern because its transcripts were detected in all tissues tested (Figure 2A). Flowers exhibited the highest transcript level, followed by stem and leaf tissues, while the roots exhibited the lowest expression (Figure 2A). This result differs from the observation that *FKF1* is predominantly expressed in soybean, *Arabidopsis*, and rice leaves.

The GFP signal exhibited predominant distribution in the nucleus and plasma membrane of tobacco epidermal cells, as depicted in Figure 2B. To confirm its nuclear residence, the nuclei of tobacco leaves were marked with a nucleus-specific dye solution (5% DAPI). Indeed, the blue fluorescence merged with the green fluorescence observed in the nucleus. Since *MsFKF1* is localized in the nucleus, we speculated whether it has transcriptional activation functions similar to those of transcription factors. To examine the transcriptional activity of the *MsFKF1* gene, we inserted the full-length coding sequence (CDS) of *MsFKF1* to the downstream of the GAL4 binding domain and introduced it into a yeast system (Y2H gold) for validation (Figure 2C,D). The results are shown in Figure 2D. Yeast transformed with the PGBKT7-*MsFKF1* plasmid failed to grow on SD/-T-H dropout plates supplemented with 3-AT compared to the positive control, indicating the absence of transcriptional activity in yeast.

### 2.3. MsFKF1 Response to Light and Showed a Circadian Rhythm Expression Pattern

**Figure 3:**
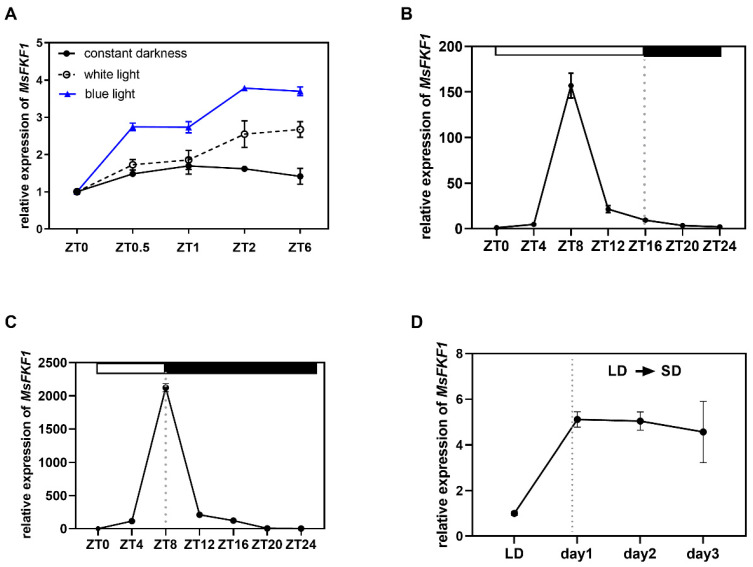
Expression pattern of the *MsFKF1* gene in response to light signal. (**A**) Expression level of *MsFKF1* in leaves in darkness, and white and blue light. (**B**,**C**) Diurnal changes in *MsFKF1* levels under LD and SD conditions. (**D**) The mRNA levels of *MsFKF1* (ZT8) in alfalfa leaves that shift from long-day to short-day. ZT, zeitgeber time (hours after dawn). The fully expanded trifoliate leaves were used in this experiment collected from 30 DAS plants. DAS, days after sowing. Values are presented as the mean value derived from three biological replicates. The bars represent the mean ± SE of three biological replicates per condition.

The analysis of gene expression showed that the expression of *FKF1* was diurnally regulated by photoperiodic in *Arabidopsis* and soybeans. A significant presence of light-responsive elements, as well as a circadian rhythm-responsive element, were identified within the promoter region of *MsFKF1* (Table S3), suggesting the potential responsiveness of the gene to light stimulation. To verify this speculation, we investigated the expression levels of *MsFKF1* under dark, white-, and blue-light conditions (Figure 3A). RT-qPCR results indicated that the level of *MsFKF1* mRNA was substantially lower under prolonged dark conditions than under illumination conditions (Figure 3A). Furthermore, under blue-light exposure, *MsFKF1* exhibited upregulated expression, with a more pronounced increase in expression than under white light. Overall, the alfalfa *MsFKF1* gene expression pattern exhibited conservation in its light response compared to many other species.

To examine whether the expression pattern of the *MsFKF1* gene in alfalfa responds to the circadian clock, the expression levels of the *MsFKF1* gene were assessed over 24 h under both long- (LD) and short-day (SD) length conditions. As expected, *MsFKF1* exhibited similar expression characteristics in both LD and SD conditions throughout the day. For instance, *MsFKF1* transcripts were abundant, peaked in the early morning (ZT8), and showed a nadir at the end of the day (ZT20). Interestingly, the short-day length increased the expression of *MsFKF1* transcripts compared to long-day conditions (Figure 3B,C and Figure S3A). This suggests that *MsFKF1* is also influenced by the photoperiod. To confirm this, we shifted LD-grown alfalfa plants to SD conditions and examined changes in *MsFKF1* expression. When plants were transferred to short-day conditions, a significant increase in expression levels was observed on the second day, which was approximately three times the expression levels observed under long-day conditions (Figure 3D).

### 2.4. Overexpression of MsFKF1 Results in Delayed Flowering in M. sativa

**Figure 4:**
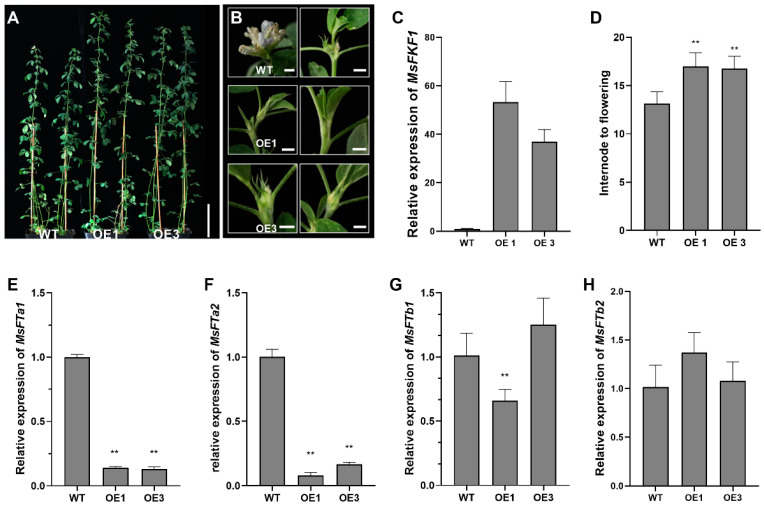
Overexpression of *MsFKF1* delays flowering time in alfalfa plants. (**A**) The image of two independent late-flowering transgenic lines (OE1, OE3) compared to WT flowering plants. Bar = 10 cm. The photo was taken one week after WT flowering. (**B**) Comparison of phenotypes of alfalfa shoot apices among WT, OE1, and OE3. Bar = 2 mm. (**C**) Expression of *MsFKF1* in WT, OE1, 3 by RT-qPCR. Leaf samples from 5-week-old plants grown under LD conditions were harvested at zeitgeber time 20 (ZT20) for RNA extraction. *MsFKF1* expression was quantified by RT-qPCR in two independent transgenic lines and WT controls. (**D**) Flowering time of two independent transgenic lines compared to WT regenerated controls. The bars indicate the mean ± SE of three biological replicates and eight clonal plants per line were analysis for flowering time assays. Asterisks represent different levels of significance (** *p* ≤ 0.01). (**E**–**H**) Expression analysis of *flowering locus T* genes in WT and OE1, 3. Fully expendable leaf samples were collected at ZT20 (peak of the *MsFTa1* gene) from the long-day condition.

To investigate whether *MsFKF1* regulates flowering in *M. sativa*, we expressed *MsFKF1* in alfalfa plants using the *35S* promoter. Due to the diurnal regulation of *MsFKF1* gene transcription, there were significant differences at different time points (Figure 3B,C). We selected the time point of 4 h after the start of light exposure (when the expression level was highest) to evaluate the expression level of *MsFKF1*. RT-qPCR results showed that the transcription level of the *MsFKF1* gene in transgenic plants increased 46- to 38.2-fold compared to the control group (Figure 4C). Subsequently, the results of the flowering time analysis showed that plants overexpressing the *MsFKF1* gene flowered 10–15 days later than WT plants (Figure 4A,B,D). Additionally, we examined the expression levels of *MsFTa1*, *MsFTa2*, *MsFTb1*, and *MsFTb2* in fully expanded leaves obtained from seedlings at ZT20 (the peak time in wild-type) (Figure 4E–H and Figure S3B). Our findings revealed significant downregulation of both *MsFTa1* and *MsFTa2* in FKF1-OE lines when compared to the WT (Figure 4E,F). We also found that the expression level of *MsFTb1* decreased only in the OE1 line (Figure 4G), while the expression level of the *MsFTb2* gene remained unchanged (Figure 4H). These results suggest that *MsFKF1* mainly delays the flowering process in alfalfa by regulating the *MsFTa1* pathway.

### 2.5. MsFKF1 Mediated the Expression of Photoperiod Pathway Genes

**Figure 5:**
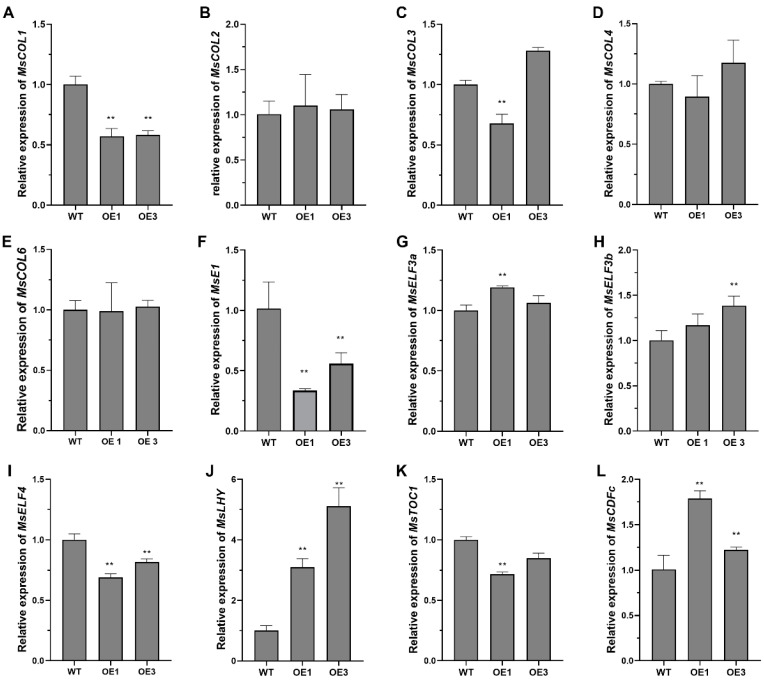
Overexpression of *MsFKF1* in alfalfa affects expression of genes related to the circadian clock. (**A**–**L**) Relative gene expression of some alfalfa *COL* genes and some key genes related to circadian rhythm in a long-day (LD) in *35S*: *MsFKF1* lines. The data included in this study were obtained from fully expanded trifoliate leaves harvested from T1 plants during day 15 at ZT4. The presented gene expression levels reflect the means of three biological replicates, and the standard error (SE) is given. Asterisks represent level of significance (** *p* ≤ 0.01). These values were normalized to *β*-actin and are presented relative to the value of the sample with the highest expression.

Previous studies have reported that the *FKF1* photoreceptor regulates the flowering process of many plants through the photoperiod pathway. To investigate the pathway of the *MsFKF1* gene in the flowering of *M. sativa*, we employed RT-qPCR to analyze the key flowering regulatory genes in *M. sativa* leaves. We demonstrated that *MsFKF1* influences the expression of the central oscillator and its related genes in the photoperiod pathway of alfalfa (Figure 5 and Figure S3C–E). For example, the overexpression of *MsFKF1* significantly increased the expression of the core gene *MsLHY* of the central oscillator compared to WT and decreased the expression of the negative feedback gene *MsTOC1* (Figure 5J,K). In addition, *MsFKF1* affected the expression of input- and output-related genes in the photoperiod pathway. We found that the expression levels of the homologous gene *MsELF3*, which inhibited flowering in *Arabidopsis* and soybeans, showed varying degrees of increase, while the expression level of the homologous gene *MsELF4* was decreased (Figure 5G–I). The RT-qPCR results of the output genes in the photoperiod pathway showed that except for *MsCOL1*, the expression of the subfamily genes in the *COL* gene family had little influence (Figure 5A–E), while the expression of the *MsCDFc* gene increased (Figure 5L), which showed negative regulation of the expression of *COL* genes in many plants. Interestingly, the expression level of the photoperiod response gene *MsE1*, unique to the Fabaceae family, was significantly decreased (Figure 5F). In general, *MsFKF1* delayed the flowering process by mediating the expression of photoperiod-associated genes in *M. sativa*.

### 2.6. MsFKF1 Increases Plant Height and Affects Forage Digestibility

**Figure 6:**
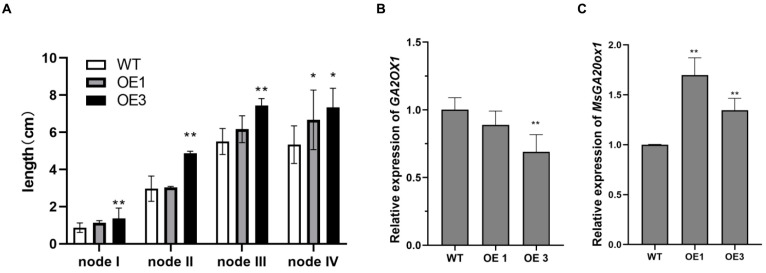
Phenotype of the WT plant compared to transgenic lines. (**A**) Data analysis of elongation stem of internode from shoots apical. (**B**,**C**) Relative gene expression of *Medicago* GA signal-related genes. Measurements were taken when the flower buds appeared during growth under long-day photoperiod at 23 °C. Bars represent the mean ±SE of 6 individual-grown clonal plants per line. Asterisks represent different significance levels (* *p* ≤ 0.05, ** *p* ≤ 0.01).

As a forage crop, the yield and quality of alfalfa are crucial factors to consider when evaluating its advantages and disadvantages. Specifically, the transgenic lines exhibited a notable increase in the plant height, leading to an overall increase in the biomass when compared to the wild-type (Table 1). The analysis of the stem node quantity and length revealed no significance different in the number of stem nodes among transgenic plants (Table 1). However, an enhancement of internode length was observed in the *MsFKF1-*OE plants (Figure 6A). Because gibberellin plays an important role in promoting plant stem elongation, we speculate that the increase in internode elongation in transgenic lines is due to the increase in gibberellin. We conducted a quantitative analysis of the key genes in the GA signaling pathway and found that the expression level of *GA20ox1* increased, while the expression level of *GA3ox1* decreased, leading to the conversion of active GA to the inactive form (Figure 6B,C and Figure S3F). Considering that reducing nondigestible and lignified tissues is a desirable trait for alfalfa biomass, we assessed the feed quality of transgenic lines with delayed flowering and compared it to the wild-type controls. The transgenic lines showed a significant increase in both neutral detergent fiber (NDF) and acid detergent fiber (ADF), as well as an increase in the lignin content, leading to decreased digestibility (Table 1).

## 3. Discussion

In *Arabidopsis*, the ZTL/FKF1/LKP2 subfamily members possess three conserved domains: the LOV domain, F-BOX domain, and tandem KELCH REPEAT domains, which are responsible for binding to key components and degradation dependent on proteases [15,16]. In addition to *LKP2*, homologs of *FKF1* and *ZTL* have been identified in the *M. sativa* genome. This difference could potentially be attributed to functional redundancy within this subfamily, leading to evolutionary divergence. Despite the similar function and conserved domain composition of genes in the *ZTL/FKF1* subfamily, evolutionary analysis indicated that *MsFKF1* is grouped with *AtFKF1* on a separate branch, suggesting functional differences between *MsFKF1* and *MsZTL* (Figure S1). Based on sequence analysis, an increasing number of *FKF1* genes have been identified in a wide range of plant species, especially in flowering plants [23]. Phylogenetic analysis revealed that the plant FKF1 protein diverges into two evolutionary branches, representing dicotyledonous and monocotyledonous plants (Figure 2A). Indeed, our analysis indicated that the plant FKF1 protein exhibits high sequence homology due to its conserved LOV, F-box, and KELCH REPEAT domains (Figure 1, Table S1), suggesting a close evolutionary distance within the plant kingdom for *FKF1*. Our observation of a consistent gene structure of *FKF1* partially supports this view (Table S2). Taken together, the identification of *MsFKF1* from alfalfa reveals a blue-light receptor that exhibits an identical gene structure and highly conserved functional domains, similar to its orthologs found in higher plants.

It appears that the nucleus blue-light receptor-encoding gene *MsFKF1* affects light-dependent signal transduction in various plant organs. Consistent with the observations that *FKF1* was mainly located in the nucleus of rice, soybean, and *Arabidopsis* [21,23,40], *MsFKF1* was found both in the nucleus and cell membrane when transiently expressed in tobacco leaves (Figure 2B) and showed no trans activity (Figure 2C,D). Expression analysis showed that ubiquitous *MsFKF1* was expressed preferentially in alfalfa flowers (Figure 2B). The leaf-preferred expression pattern was observed for *GmFKF1*s in soybean [22], *AtFKF1* in *Arabidopsis* [14], *SlFKF1* in tomato [24], and *OsFKF1* in rice [21], while they all showed lower expression in flowers. This difference suggests that the *MsFKF1* gene may be involved in floral organ development, and the expression levels in various organs of flowers need further confirmation. *MsFKF1* can respond to light induction to some extent, especially blue light (Figure 3A), which is consistent with reports in *Arabidopsis thaliana* [14], tomato [24], and rice [21]. Proteins belonging to the ZTL/FKF1/LKP2 family are known to play roles in blue-light photoreception and the modulation of downstream responses, including the regulation of the circadian clock and photoperiodic flowering time [14,41,42]. Similarly, whether in LD or SD conditions, we found that *MsFKF1* showed a robust cycling expression pattern throughout the day, reaching its peak at ZT8, except that SD conditions significantly increased the expression level. These results are in partial agreement with reports in soybean, rice, and *Arabidopsis* [20,21,22,32]. Furthermore, the induction of *MsFKF1* expression at ZT8 was reversed after the plants were returned to SD (Figure 3D), which could account for its photoperiodic response. In addition, the fact that the peak time of *FKF1* mRNA in alfalfa (ZT8) and soybeans (ZT10) is not affected by day-length compared to rice and *Arabidopsis* may be unique to legumes.

*FKF1* is a key regulator of plant photoperiod-dependent flowering and developmental processes. In *Arabidopsis* and rice, light-dependent *FKF1* binds to and degrades the *CDF* negative regulatory transcription factor, thereby promoting the transcription level of the *CO/Ehd1* transcription factor to promote early flowering [21,32]. Recent soybean research shows that a unique pathway of *GmFKF1*s suppressing flowering time is genetically dependent on depressing legume-specific *E1* family genes [22]. Similar to soybeans, we showed that *MsFKF1* inhibited the transcription of *MsE1* and *MsCOL1* genes and thus depressed *MsFTa1*, thereby suppressing the flowering process of alfalfa (Figure 4 and Figure 5). Furthermore, it is possible to modulate flowering in alfalfa via the regulation of the circadian rhythm pathway. In legume plants, researchers revealed that the expression of clock-associated genes, including *LHY*, *TOC1*, and the *EC* complex, were implicated in regulating the flowering time [43,44,45,46]. In this study, expression analysis of *MsFKF1*, a gene showing diurnal oscillation and light-responsive characteristics, provided evidence to support this perspective (Figure 3B–D). Moreover, the constitutive expression of *MsFKF1* results in a significant increase in the expression levels of *MsLHY* and *MsCDFc* (Figure 5), which negatively regulate flowering in *Medicago* [43,47]. Taken together, we propose that the FKF1–circadian clock–E1 pathway may have a significant impact on the photoperiodic flowering pathway in alfalfa, diverging from the CO-centric pathways observed in *Arabidopsis* and rice. More detailed work on the relationship between *MsFKF1* and *MsLHY*, including transcriptional regulation, protein interaction, and genetic relationships, needs to be further confirmed.

In alfalfa, plant height is commonly considered a core yield-related trait in breeding and is determined by the number and length of internodes [48,49]. Gibberellins (GAs) are crucial for regulating diverse biological processes associated with plant growth and development, including flowering and plant height [50]. Recently, studies have reported the association of specific genes in GA metabolic pathways with plant height in alfalfa. One example is the gene *MSD1*, which encodes the GA20ox1 enzyme responsible for gibberellin 20-oxidase activity. Reduced levels of endogenous GA in mutant *msd1* result in dwarfism in *M. truncatula* [48]. However, few studies have explored the molecular mechanisms governing plant height and internode length regulation in alfalfa. In this study, we observed that the overexpression of *MsFKF1* resulted in increased plant height, which was subsequently investigated by analyzing the internode length (Table 1 and Figure 6A). Additionally, we obtained the mRNA levels of essential genes involved in the GA metabolic pathway, such as *GA20OX1* (*MsSD1*) and *MsGA2OX1*, in both the *MsFKF1* transgenic lines and wild-type (WT) plants (Figure 6B,C). Our results showed an increase in the expression of the *MsSD1* gene in the *MsFKF1* transgenic lines, whereas the expression of the *MsGA2OX1* homolog gene remained largely unchanged. It is hypothesized that *MsFKF1* regulates GA-synthesis-pathway-related genes to promote plant height in alfalfa plants.

## 4. Materials and Methods

### 4.1. Plant Materials and Growth Conditions

*M. sativa* cv. Zhongmu No. 4, developed in our laboratory (Institute of Animal Science, Chinese Academy of Agricultural Sciences), was used in this study. The seeds were sowed in moist pot for 5 days and grew in a substrate (nutrient soil: vermiculite: perlite = 1:2:1) or in Hoagland’s solution in green house (23 °C; 16 h light/8 h darkness). For the tissue expression pattern analysis, 45-day-old plant tissues of leaves, stem segments, flowers, buds, and roots were harvsted, respectively, in this experiment. For light-quality treatment, on day 30, one of the three plants was moved into continuous darkness, and the other was moved to white and blue light. In the experiment, treatments of 0.5, 1, 2, and 6 h were conducted. At each time point, mature leaves from different growth-condition seedlings were harvested separately and immediately frozen in liquid nitrogen.

### 4.2. RT-qPCR

Total RNA was extracted according to the instructions of Promega’s Plant Total RNA Extraction Kit and evaluated by NanoDrop. For the first-strand cDNA synthesis, one microgram of total RNA was used for 1st-strand cDNA reverse transcription using a cDNA Synthesis Kit (Takara, Dalian, China). RT-qPCR analysis was performed using SYBR Premix Ex Taq (TaKaRa, Dalian, China) on the CFX96^TM^ System (Bio-Rad, Hercules, CA, USA). *MsACTIN2* and *AtACIN2* (At3g18780) were used as internal reference genes for *M. sativa* and *Arabidopsis*, respectivly.

### 4.3. Isolation and Phylogenetic Analysis of Alfalfa MsFKF1 Homologs

In order to obtain FKF1 homologs from alfalfa, AtFKF1 (At1g68050) was used as a query sequence to search the *M. sativa* genome (https://figshare.com/s/fb4ba8e0b871007a9e6c, accessed on 1 October 2022) [51]. For the phylogenetic analysis, MEGA version 6.0 [52] was used. In addition, multiple sequence alignment was performed using DNAMAN version 8.0 (Lynnon Corporation, Vaudreuil-Dorion, QC, Canada). InterPro (http://www.ebi.ac.uk/interpro/search/sequence/, accessed on 1 October 2022) was used to analyze the protein signature of MsFKF1.

### 4.4. Plasmid Construction and Transgenic Alfalfa Generation

We utilized degenerate primers (Table S4), designed based on the sequence of the *MsFKF1* genes coding sequence (CDS), for the amplification and sequenceing process. The full-length CDS of *MsFKF1* removed the stop codon was recombined into the pCamba1302 plasmid vector. Subsequently, the constructed plasmid was transformed into *Agrobacterium* (EHA105) utilizing the freeze–thaw method [53]. We utilized the *Agrobacterium*-mediated leaf transformation method of alfalfa [54] to obtain transgenic regenerated seedlings.

### 4.5. Plant and Flowering Time Assays with Alfalfa

To determine the flowering time, OE lines and the controls of alfalfa were grown in a greenhouse (23 °C, 10 h light/14 h dark) for at least one month. The plants were then counted from the apex to the base, and the portion below the fourth node was cut from the stem, and each node was separated and placed for rooting in vermiculite. Once rooted, the individual cuttings were transferred to pots with substrate (soil:perlite:vermiculite = 3:1:1) and were further cultivated in the greenhouse. The cuttings that developed into plants were shifted to an inductive photoperiod (16 h light/8 h dark) to induce flowering. The flowering time was based on the time of the first flower bud. Flowering days, internode length, fresh weight, and plant height were recorded for each condition.

### 4.6. Subcellular Localization and Transactive Assay

For subcellular localization, the epidermal cells of 5-week-old tobacco seedlings were analyzed using an inverted fluorescence microscope, as previously described [55]. To assess the transcriptional activation activity in the yeast, we subcloned the full-length coding sequence of MsFKF1 into the pGBKT7 vector to fuse with the GAL4 binding domain, and then introduced to the yeast (Y2H gold) through an electric shock system with the parameters of 1.5 KV and 200 Ω. Transformants were selected on a tryptophan-lacking SD medium (SD/-T), followed by growth on an SD medium lacking tryptophan and histidine (SD/-T-H) with 15 mM 3-AT for 3 days to evaluate the transcriptional activation activity based on the growth status.

### 4.7. Determination of the Forage Quality Assays

To determine the forage quality, the samples were analyzed following the method described by Lorenzo C.D. in 2020 [4]. The above-ground biomass was harvested at the early-flowering stage after the first cut and dried for 4 days in a 70 °C oven before being ground using a grinder. A near-infrared reflectance spectroscopy system (NIRS 6500 Foss, Silver Springs, MD, USA) was used to measure the quality of the forage.

### 4.8. Statistical Analysis

Significant differences were determined using Student’s *t*-test with R 4.3.1 software.

## 5. Conclusions

In this study, we isolated a blue-light receptor family member named *MsFKF1*, which showed upregulation in response to light stimulation, particularly sensitive to blue light. Further analysis suggested that the transcriptional expression of *MsFKF1* was influenced by circadian rhythms, peaking after 8 h of light exposure, with an additional increase in the expression under short-day photoperiods. The MsFKF1 protein was found to be localized in the nucleus and cell membrane, but had no transcriptional activity in yeast. According to the results of the functional character assay, we found that the overexpressing of *MsFKF1* in alfalfa delayed the flowering time and increased the biomass by the elongation of the node length in a long day, significantly altering the transcript expression levels of the photograph flowering time marker genes, including *MsFTa1*, *MsE1*, and *MsLHY*. Our findings provide genetic resources for flowering time regulation and yield improvement in the molecular breeding of alfalfa.

## Figures and Tables

**Table 1:** Overexpression of *MsFKF1* alters plant height and forage quality.

Genotype	Plant Height (cm)	Stem NO.	Fresh Weight (g)	% ADF	% NDF	% Lignin	% Digestibility
WT	54.70 ± 9.66	21.60 ± 2.19	4.74 ± 0.62	38.01 ± 0.16	51.40 ± 0.18	7.94 ± 0.03	74.52 ± 0.21
OE1	62.31 ± 6.89 **	21.29 ± 2.06	5.56 ± 1.79 **	41.76 ± 0.31 **	56.83 ± 0.24 **	8.68 ± 0.09 **	70.76 ± 0.31 **
OE3	61.35 ± 6.03 **	21.38 ± 1.51	5.92 ± 0.78 **	42.96 ± 0.07 **	57.23 ± 0.12 **	8.69 ± 0.02 **	69.81 ± 0.11 **

ADF and NDF are abbreviations for acidic detergent fiber and neutral detergent fiber, respectively. Asterisks represent different significance levels (** *p* ≤ 0.01).

## Data Availability

Data are contained within the article and Supplementary Materials.

## Supplementary Materials

The following supporting information can be downloaded at: https://www.mdpi.com/article/10.3390/plants13050655/s1, Figure S1. Phylogenetic analysis of blue light receptor proteins of *Medicago* and *Arabidopsis*. A phylogenetic tree was generated using the maximum likelihood method. FKF1 showed greater similarity to ZTL and LKP2 compared to the other four blue-light receptors (CRY1, CRY2, PHOT1, and PHOT2); Figure S2. Phylogenetic analysis of MsFKF1 and its orthologs. The phylogenetic tree was generated through the utilization of the neighbor-joining method, following sequence alignment conducted with the Clustal W program. The branch numbers present on the tree represent the percentage of replicates that provide support for each specific branch, as determined by implementing the bootstrap method with 1000 replicates. The scale bar depicted on the tree corresponds to a measurement of 0.05 amino acid substitutions per residue. The accession numbers of the proteins employed in constructing the phylogenetic tree can be found in Table S2; Figure S3. The melting curve diagram of RT-qPCR primers used in this study. (A) The melting curve diagram of the *MsFKF1* primer is used to analyze the effect of photoperiod on the expression level of *MsFKF1*. (B) The melting curve diagram of the RT-qPCR for alfalfa *flower locus T* gene primers. (C-E) The melting curve diagram of the RT-qPCR for Flowering time related genes primers. (F) the melting curve diagram of the RT-qPCR for GA biosynthesis related genes primers; Table S1. Sequence homology of FKF1 proteins in the indicated species; Table S2. Comparison of the exon/intron composition of *FKF1* in the indicated species; Table S3. Cis-element of the *MsFKF1* promoter; Table S4. Primers used in this study.
