# Supplementary material for: Functional Characterization of the MsFKF1 Gene Reveals Its Dual Role in Regulating the Flowering Time and Plant Height in Medicago sativa L
Source: Plants (Basel). 2024 Feb 27;13(5):655. doi: 10.3390/plants13050655 (PMC10934225; doi:10.3390/plants13050655)
Supplement: Supplementary file 1 [file plants-13-00655-s001.zip › plants-2811860-supplementary.pdf]

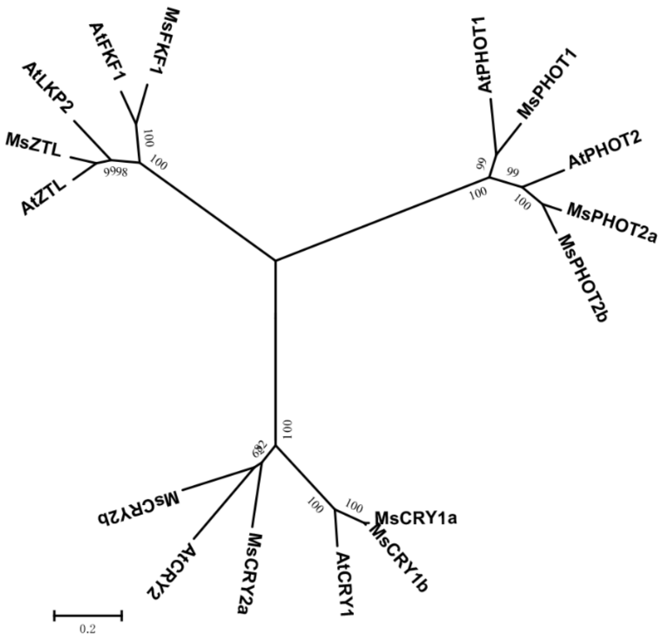

Figure S1. Phylogenetic analysis of blue light receptor proteins of *Medicago* and *Arabidopsis*. A phylogenetic tree was generated using the maximum likelihood method. FKF1 showed greater similarity to ZTL and LKP2 compared to the other four blue-light receptors (CRY1, CRY2, PHOT1, and PHOT2).

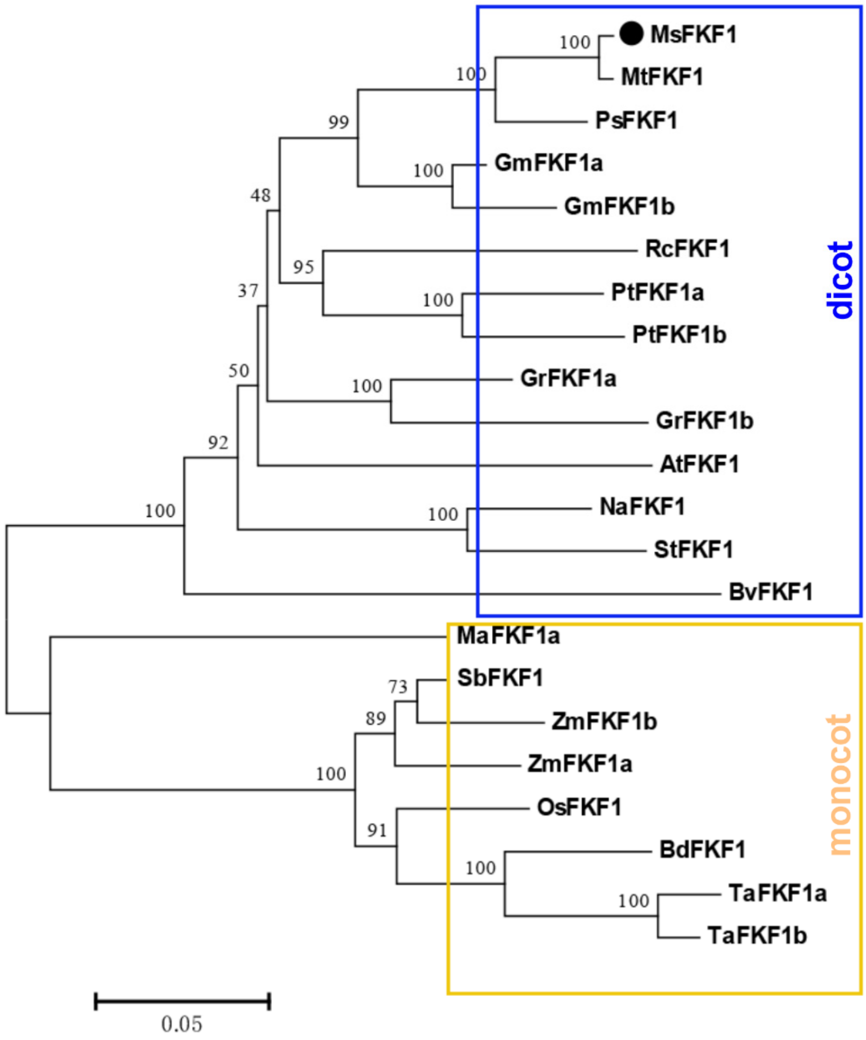

Figure S2. Phylogenetic analysis of MsFKF1 and its orthologs.
The phylogenetic tree was generated through the utilization of the neighbor-joining method, following sequence alignment conducted with the Clustal W program. The branch numbers present on the tree represent the percentage of replicates that provide support for each specific branch, as determined by implementing the bootstrap method with 1000 replicates. The scale bar depicted on the tree corresponds to a measurement of 0.05 amino acid substitutions per residue. The accession numbers of the proteins employed in constructing the phylogenetic tree can be found in Table S1.

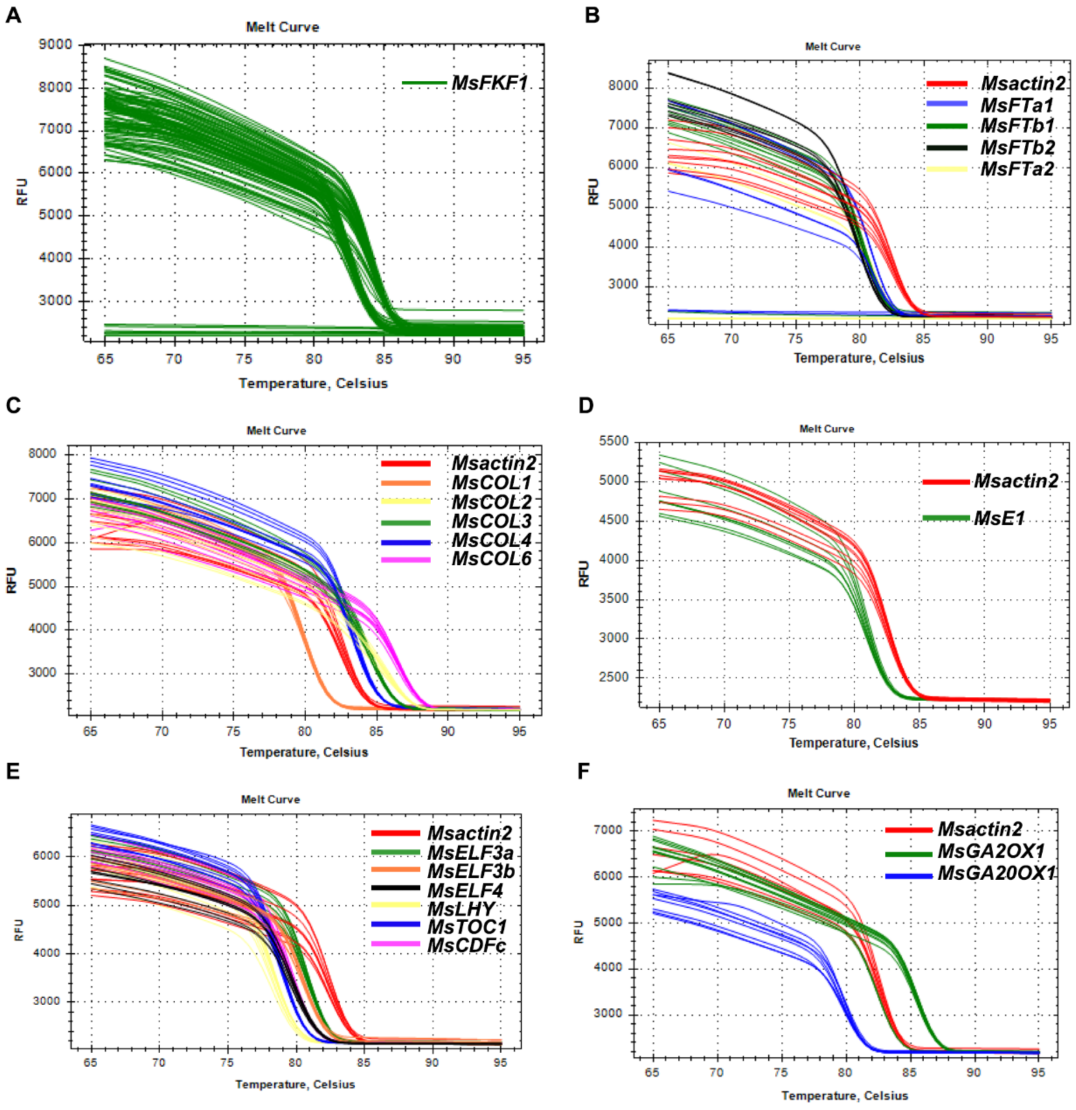

Figure S3. The melting curve diagram of RT-qPCR primers used in this study.

(A) The melting curve diagram of the MsFKF1 primer is used to analyze the effect of photoperiod on the expression level of MsFKF1. (B) The melting curve diagram of the RT-qPCR for alfalfa *flower locus T* gene primers. (C-E) The melting curve diagram of the RT-qPCR for Flowering time related genes primers. (F) the melting curve diagram of the RT-qPCR for GA biosynthesis related genes primers.

Table S1. Sequence homology of FKF1 proteins in the indicated species.

Gene	Homology matrix
MsFKF1	100%
MtFKF1	99.3% 100%
GmFKF1a	90.4% 90.2% 100%
AtFKF1	81.5% 81.5% 82.8% 100%
ZmAGO3	72.9% 72.7% 73.2% 71.2% 100%

Table S2. Comparison of the exon/intron composition of FKF1 in the indicated species.

Species	Gene symbol	Accession numbers	CDS (bp)	amino acid (a.a.)	extron 1 length (bp)	intron length (bp)	extron 2 length (bp)
Medicago sativa	*MsFKF1*	*Msa0930760*	1911	636	384	677	1942
Medicago truncatula	*MtFKF1*	*MTR_8g105590*	2328	635	384	677	1942
Pisum sativum	*PsFKF1*	*Psat7g007600*	2453	610	447	359	2004
Glycine max	*GmFKF1a*	*Glyma05g239400*	2400	620	517	992	1883
	GmFKF1b	*Glyma08g046500*	2252	647	377	1040	1875
Rosa chinensis	*RcFKF1*	*RchiOBHm_Chr4g0442801*	2354	631	464	822	1888
Nicotiana attenuata	*NaFKF1*	*A4A49_16690*	2307	634	326	4548	1981
Gossypium raimondii	*GrFKF1a*	*B456_002G166800*	2392	629	416	1892	1974
	GrFKF1b	*B456_008G168500*	2301	627	376	704	1923
Arabidopsis thaliana	*AtFKF1*	*At1g68050.1*	2168	619	369	100	1797
Beta vulgaris	*BvFKF1*	*BVRB_6g154770*	2322	633	490	5653	1832
Populus trichocarpa	*PtFKF1a*	*Potri.010G105700.v4.1*	2442	627	300	411	3240
	PtFKF1b	*Potri.008G135200.v4.1*	2532	637	454	1147	2076
Solanum tuberosum	*StFKF1*	*PGSC0003DMT400051416*	2239	634	343	2358	1896
Sorghum bicolor	*SbFKF1*	*SORBI_3005G145300*	2111	594	197	1785	1914
Zea mays	*ZmFKF1a*	*GRMZM2G107945*	2582	629	494	8694	2086
	ZmFKF1b	*GRMZM2G106363*	2367	618	422	743	1945
Triticum aestivum	*TaFKF1a*	*TraesCS4A02G164000*	2320	626	437	862	1883
	TaFKF1b	*TraesCS4B02G157500*	2191	620	300	843	1891
Brachypodium distachyon	*BdFKF1*	*KQJ88263*	2330	621	423	1529	1907
Oryza sativa	*OsFKF1*	*Os11g34460*	2190	630	397	2651	1791
Musa acuminata	*MaFKF1a*	*Ma07_g10990*	2358	616	519	1609	1839

Table S3. Cis-element of the MsFKF1 promoter.

Name	Cis element	Number	Description
circadian	CAAAGATATC	1	circadian control
GT1-motif	GGTTAA	2	light responsiveness
G-box	CACGA/TC	3	light responsiveness
TCT-motif	TCTTAC	1	light responsiveness
ATCT-motif	AATCTAATCC	2	light responsiveness
Box 4	ATTAAT	1	light responsiveness
GATA-motif	GATAGGA	1	light responsiveness
ABRE	ACGTG	2	abscisic acid responsiveness
CGTCA-motif	CGTCA	3	MeJA-responsiveness
TGACG-motif	TGACG	3	MeJA-responsiveness
CAT-box	GCCACT	2	related to meristem expression

Note: *The MsFKF1* promoter sequence was extracted from 2000 bp upstream of the start codon (ATG) in the assembled genome of Zhongmu No. 4.

Table S4. Primers used in this study.

Name	Popups	Forward (5' → 3')	Reverse (5' → 3')
MsFKF1	clone	GAAAATTATTGTACACTCCACGG	CGGTTCAGTTGATTTTCATAAGT
Homo-FKF1	Overexpression and GFP	CTCTAGAGTTAACCGGGCTCAG ATGTGTGGCATTCTTCAGCT	CCTCAGAAATCAACTTTTGCTC CATGTCAGAGTCTTGTCG
BD-FKF1	Assemble clone to PGBKT7	atatggccatggaggccgaattcATGTGTGG CATTCTTCAGCT	aggtcgacggatccccgggaattTCACATG TCAGAGTCTT
qMsFKF1	qPCR	CAGGTCTTCATTCACCGTCTC	ACTCATTCAGCACCCATTCC
qMsFTa1	qPCR	TCAGAAACCTCAACACCCTAGTT A	CCTGGAATATCAGTCACCAACC AG
qMsFTa2	qPCR	ACCCAACAACCTTCTACACCCTG	AATCCCTAAATTGGGTCGTGGCT
qMsFTb1	qPCR	GACTACAGGGACAACTTTCGGAG	GCCATCCAGGAGCTAATATAGTG A
qMsFTb2	qPCR	GGGGACAACGTTCGGAC	CGCCATCCTGGAGCTAATATATT G
qMsE1	qPCR	TCTTGTGATGAAGCCTCTAC	TATCCCTTGTTGTCGTTGT
qMsCOL1	qPCR	CTTGGGTTGGAGTTTGAGTC	TGTTCCTTTTGGAGGTCTTG
qMsCOL2	qPCR	TCTCCAAACTCCTTCAAACC	TACAACTCCAACCTCCATCG
qMsCOL3	qPCR	GAATCCAACACTTCTCACTCTG	TCCTCTTCTCCCTATACCTCA
qMsCOL4	qPCR	GGTGTGAATAGTAACCAAGGGAT	CATAGATGAAGGAACTGCGAGA
qMsCOL6	qPCR	TTTCCGTGTGACTATTGCG	AAAGATGAAGAGGTGGGGTT
qMsELF4	qPCR	AGTTTCAGGCAGGTTCAGTCG	CTTCACCATATTATCAGGCATTCT
qMsELF3a	qPCR	AAAGGGATCTAAACAATCTGCG	TTGGCTTGTCTTACTGAGGTGTC
qMsELF3b	qPCR	CCAGCGAGGCAAGCTAAGAA	CCATTGTAGTGTCAAGGAGGC
qMsLHY	qPCR	GAGGAGCATAAAGATGAGGAAA G	CCGAAGATACAGATGAACAAGG
qMsTOC1	qPCR	AGCAAGAGTGGTGATGGATTCA	TGCCGTGCGGATTTTACAGA
qMsCDFc	qPCR	CTTCATCTCCCTCTTCTTCTCCA	AGGCTCCATCGTCTTGTGCT
qMsGA20ox1	qPCR	GAATGAGCCTTGGAGTTGG	TGGAATGCCATTGATCGTC
qMsGA2ox1	qPCR	TCCCCTGCTTTTGGTAAAGAT	GGACAAGGGGGATAATGGTTG
